# Spatiotemporal Correlations between Blood-Brain Barrier Permeability and Apparent Diffusion Coefficient in a Rat Model of Ischemic Stroke

**DOI:** 10.1371/journal.pone.0006597

**Published:** 2009-08-11

**Authors:** Saeid Taheri, Eduardo Candelario-Jalil, Eduardo Y. Estrada, Gary A. Rosenberg

**Affiliations:** Department of Neurology, University of New Mexico, Health Sciences Center, Albuquerque, New Mexico, United States of America; Julius-Maximilians-Universität Würzburg, Germany

## Abstract

Variations in apparent diffusion coefficient of water (ADC) and blood-brain barrier (BBB) permeability after ischemia have been suggested, though the correlation between ADC alterations and BBB opening remains to be studied. We hypothesized that there are correlations between the alteration of ADC and BBB permeability. Rats were subjected to 2 h of transient middle cerebral artery occlusion and studied at 3 and 48 h of reperfusion, which are crucial times of BBB opening. BBB permeability and ADC values were measured by dynamic contrast-enhanced MRI and diffusion-weighted imaging, respectively. Temporal and spatial analyses of the evolution of BBB permeability and ADC alteration in cortical and subcortical regions were conducted along with the correlation between ADC and BBB permeability data. We found significant increases in BBB leakage and reduction in ADC values between 3 and 48 h of reperfusion. We identified three MR tissue signature models: high K_i_ and low ADC, high K_i_ and normal ADC, and normal K_i_ and low ADC. Over time, areas with normal K_i_ and low ADC transformed into areas with high K_i_. We observed a pattern of lesion evolution where the extent of initial ischemic injury reflected by ADC abnormalities determines vascular integrity. Our results suggest that regions with vasogenic edema alone are not likely to develop low ADC by 48 h and may undergo recovery.

## Introduction

The use of magnetic resonance imaging (MRI) techniques, including apparent diffusion coefficient (ADC) obtained by diffusion-weighted imaging (DWI), perfusion-weighted imaging (PWI), and contrast-enhanced MRI has been increasingly important in evaluating various treatment strategies in both experimental models of cerebral ischemia [1]–[4] and stroke patients [5]–[10]. Advances in imaging speed and computer analysis permit multimodal assessments of individual pixels, which improve predictions of tissue outcome.

Blood-brain barrier (BBB) disruption is an important pathological hallmark of ischemia with or without reperfusion, and it is associated with vasogenic edema, hemorrhagic transformation, and has been linked to poor outcome in stroke patients [8], [9]. Using either the ^14^C-Sucrose quantitative method or the extravasation of Evans blue-albumin, others and we had previously described a biphasic opening of the BBB in the rat middle cerebral artery occlusion (MCAO) stroke model [11]–[13]. There is an early significant increase in BBB opening after 2–3 h of reperfusion following MCAO, the timing of which depends on the duration of ischemia [14]. After this initial opening, the BBB restores its functions and no significant changes in permeability to either ^14^C-sucrose or Evans blue-albumin are observed until 24 to 48 h after reperfusion. Dramatic BBB breakdown occurs after 48 h of recirculation, which is accompanied by significant vasogenic edema and leukocyte infiltration [11]–[13].

Previous studies have reported an associated decline in ADC intensity in ischemic lesion induced by MCAO in animal models [15], [16]. Although there is not a strong consensus on the origin of the decline in ADC in ischemic lesions, swelling of cells and limiting the intracellular space are plausible explanations for the reduction of ADC of water. Reperfusion after focal cerebral ischemia leads to a regional disruption of the BBB and vasogenic edema [14], [17]. Regional changes in BBB permeability and ADC after stroke have been associated with different pathophysiological alterations in the lesion area [17]–[19]. However, the correlation between the alteration of ADC and the BBB permeability has been incompletely characterized. Development of an *in vivo* method of BBB permeability quantification using fast T1 sequences and multiple times sampling after contrast injection has made it possible to perform pixel-by-pixel measurement of permeability coefficient and ADC. It has been previously shown that MRI-based BBB quantification using Gd-DTPA highly correlates with the ^14^C-sucrose method to quantify BBB breakdown in rat focal cerebral ischemia [20], [21].

We hypothesize that there are regional correlations between the edema represented by reduced ADC and BBB integrity disruption. We first compared the temporal changes in BBB permeability in cerebral cortex and subcortical regions after the induction of focal cerebral ischemia in a rat model. We next determined whether ADC reduction correlated with BBB permeability changes in different brain areas. Cerebral cortex and subcortical regions were studied at 3 and 48 h of reperfusion, representing the early and delayed BBB disruption, respectively. Quantitative spatial and temporal information about the blood-to-brain influx rate constants (K_i_) was estimated from a series of dynamic contrast-enhanced magnetic resonance images (D-CEMRI) [22]–[25]. ADC values were calculated from images obtained by DWI. Using generalized linear modeling techniques, we show a region-specific lesion evolution where the extent of initial ischemic injury reflected by low values in ADC maps determines BBB permeability changes.

## Results

**Figure 1:**
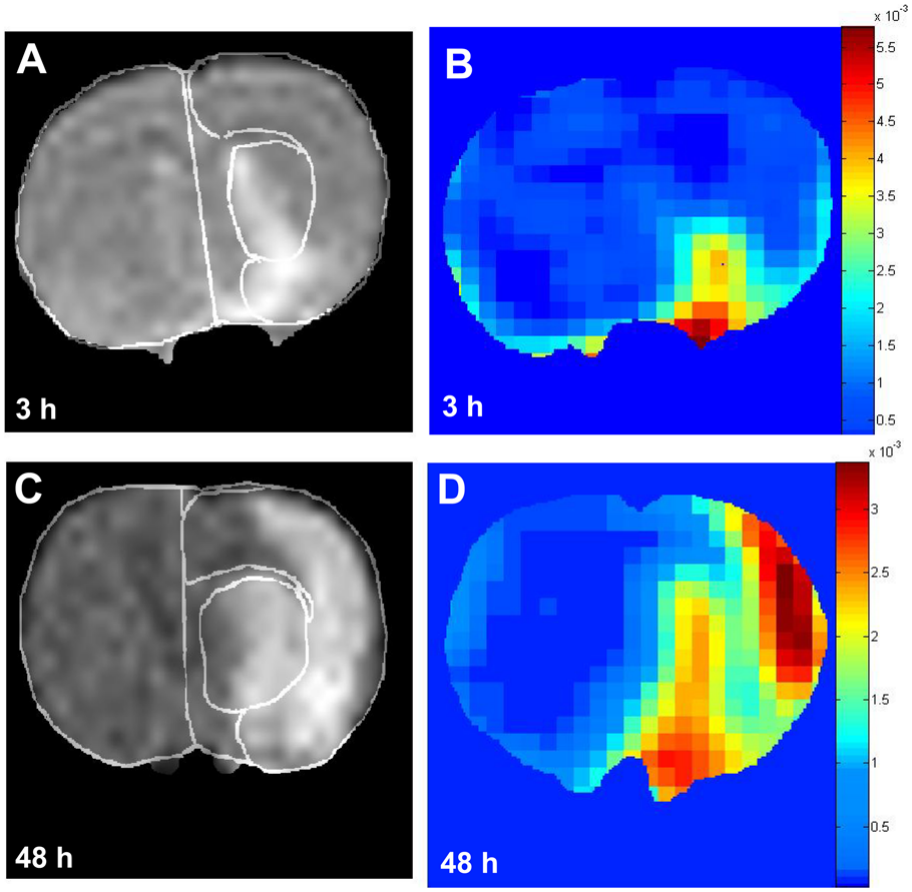
Representative DWI (A and C) and corresponding permeability maps (B and D) of coronal sections of rat brains. Images shown are from two different groups: A and B from group 1 (scanned at 3 h, n = 8) and C and D from group 2 (studied at 48 h, n = 8). DWI images were used to calculate ADC maps and generate tissue signature maps shown in Fig. 5A and 5B. Areas of the subcortex and cerebral cortex are demarcated in A and C.

Marked regional differences were observed in the degree of increment in BBB permeability and reduction in ADC values (calculated from DWI images) in cerebral cortex and subcortical regions at 3 and 48 h following 2 h of MCAO. The extent of the lesion calculated based on the altered ADC and BBB permeability was larger at 48 h compared with 3 h in cerebral cortex and subcortical areas. **Figure 1** shows representative DWI and BBB permeability maps at 3 and 48 h of reperfusion. As expected, lesion size at 3 h after the reperfusion is smaller than at 48 h. Moreover, DWI images show that the lesion at 3 h is confined to the subcortical and partly to cortical areas (mainly piriform cortex), while at 48 h of recirculation the lesion extends to large areas of the cerebral cortex and subcortex (**Fig. 1A and 1C**). Based on ADC maps obtained from DWI, the progression of lesion is from the subcortical area towards the cortical area.

Permeability maps show dramatic increases in areas with significant BBB leakage between 3 and 48 h of reperfusion in both cerebral cortex and subcortex (**Fig. 1B and 1D**). However, at the earlier time point (3 h) the increased permeability was confined to the piriform cortex and small areas of the striatum (**Fig. 1B**). Using ^14^C-sucrose to evaluate BBB disruption, previous studies have shown that the piriform cortex is the main site in early BBB injury [12], [26].

**Figure 2:**
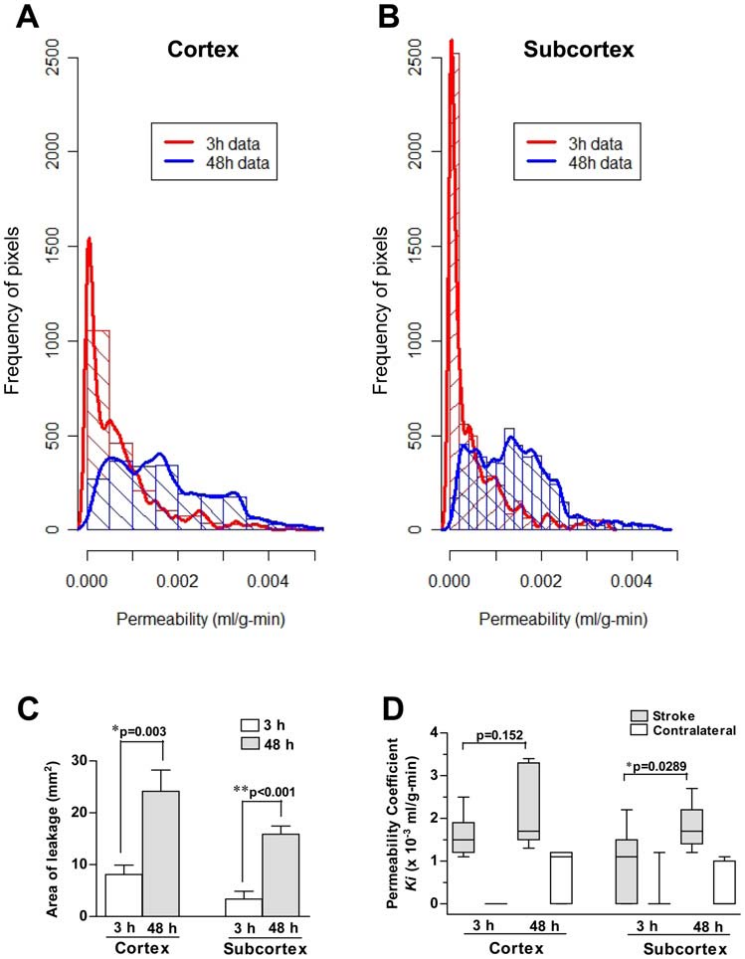
Cumulative spatiotemporal distribution of BBB permeability for cerebral cortex and subcortex (Panels A and B). Statistical analysis of raw data presented in A and B was performed by calculating the area of leakage for each brain region with K_i_ values higher than 0.001 ml/g-min. As shown in panel C and D, BBB permeability changes at 48 h were significantly higher than at 3 h. The vertical axis shows the frequency of pixels with different K_i_ values (shown in the horizontal axis).

The spatiotemporal distribution of BBB permeability for cerebral cortex and subcortical areas is presented in **Fig. 2**. The BBB permeability at 3 h of reperfusion is not as high as the permeability at 48 h for both cerebral cortex and subcortical areas, as shown by the area under the curve (**Fig. 2A and 2B**). There was a marked increase in the percentage of pixels with K_i_ higher than 0.001 ml/g-min between 3 and 48 h of recirculation (24.5% at 3 h *vs.* 68.4% at 48 h for cerebral cortex, p<0.0001, χ^2^ test; and 17% at 3 h *vs.* 64.2% at 48 h for the subcortical regions, p<0.0001, χ^2^ test). In a rat model of focal cerebral ischemia, it has been previously shown that K_i_ values higher than 0.001 ml/g-min are abnormal [27]. We calculated the area of leakage by multiplying the number of pixels with K_i_ values higher than 0.001 ml/g-min by the pixel size. Quantitative data shown in **Fig. 2C** indicate that there is a statistically significant (p<0.01) increase in the area of leakage in the ipsilateral cortex and subcortex between 3 and 48 h of reperfusion. For each brain region, we calculated the median of K_i_ values for ipsilateral (stroke) and contralateral side and found a significant increase in the BBB permeability values between the ischemic and contralateral side at a given time point (**Fig. 2D**). However, a significant increase (p<0.03) in the K_i_ values was only found for the subcortex between 3 and 48 h of reperfusion. There was a trend toward an increase in the K_i_ values at 48 h with respect to 3 h in the cerebral cortex, but this increase did not reach statistical significance (**Fig. 2D**).

**Figure 3:**
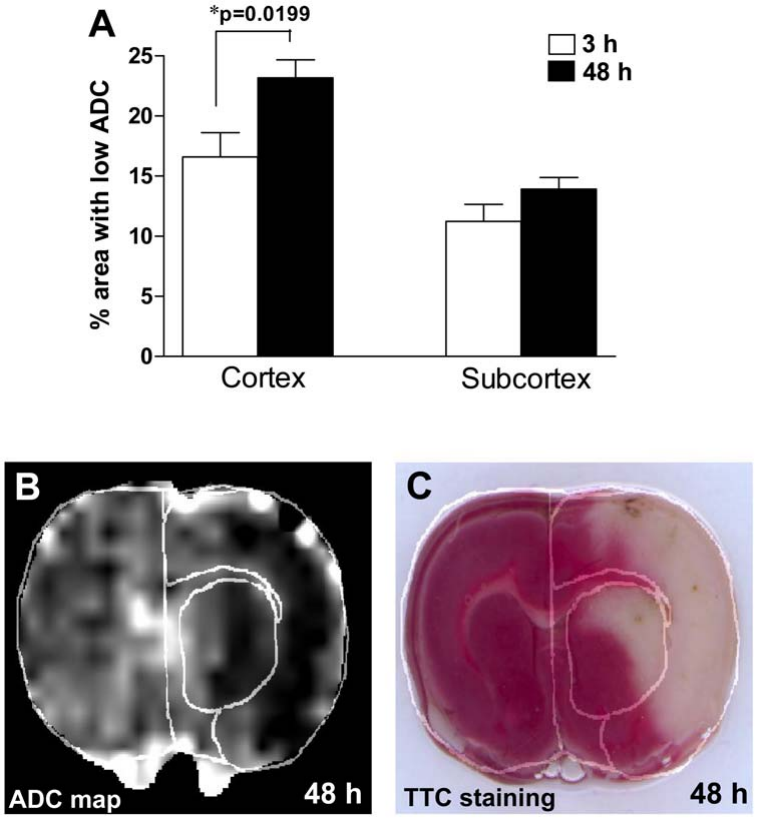
A. Changes in the area with hypointensive ADC at 3 and 48 h of reperfusion. Significant increases in areas with ADC abnormalities were found between 3 and 48 h in the cerebral cortex. Brain damage observed at 48 h in ADC maps (B) was confirmed histologically using TTC staining (C). Areas of the subcortex and cerebral cortex are demarcated in panels B and C.

We calculated the ipsilateral area with ADC values less than 80% of the mean contralateral hemisphere values on ADC maps for each experimental animal at 3 and 48 h of recirculation. As expected, we found larger areas with ADC reduction at 48 h as compared with 3 h in the cerebral cortex (**Fig. 3**). However, we did not find a significant increase in areas with low ADC between 3 and 48 h in the subcortex (**Fig. 3A**). Tissue damage observed at 48 h in ADC maps (**Fig. 3B**) was confirmed by histological staining using 2,3,5-triphenyltetrazolium chloride (TTC) (**Fig. 3C**).

**Figure 4:**
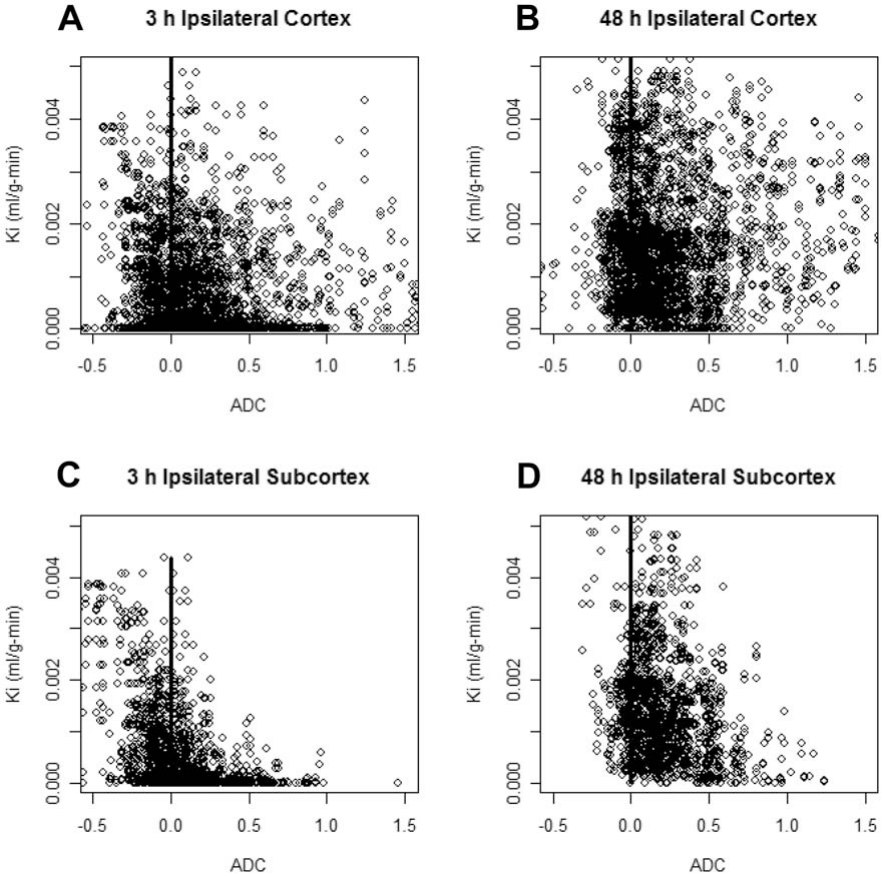
Scatterplots and fit lines showing the correlations between ADC and K_i_ for the ipsilateral side of cerebral cortex (A and B) and subcortex (C and D) at 3 and 48 h of reperfusion following 2 h of MCAO in the rat.

**Figure 5:**
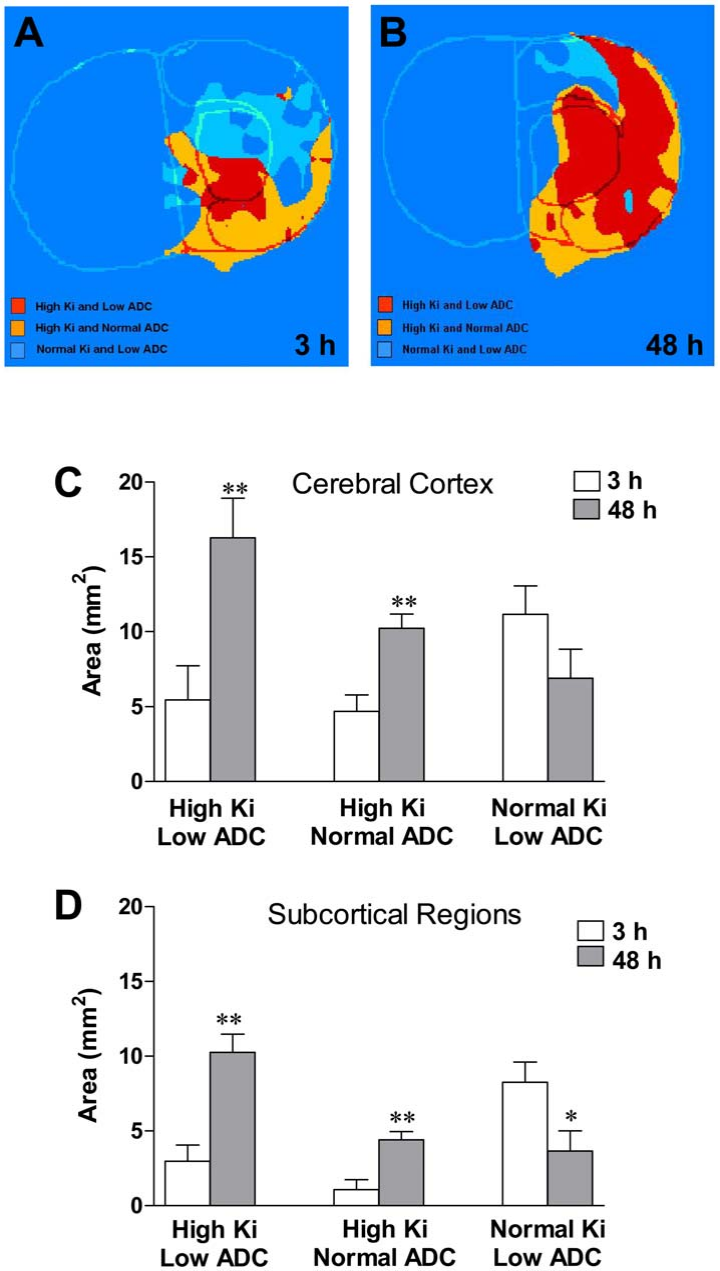
Analysis of correlations between BBB permeability and the ADC values in cerebral cortex and subcortex. Color-coded K_i_-ADC maps (A and B) show the three main populations of pixels with different K_i_ and ADC values for both time points. Quantitative changes of each area over time are presented in panels C and D. Based on the abnormality of ADC values and the BBB permeability; we have recognized three different areas in the ipsilateral side. These areas are labeled as tissue signatures corresponding to different pathophysiology in the evolution of lesion. Each tissue signature is represented by a different color at two different time points, 3 and 48 h of reperfusion. *p<0.05 and **p<0.01 with respect to 3 h.

Changes in ADC reflect both a decrease due to cytotoxic edema and an increase from vasogenic edema, which may be related to BBB permeability changes. Thus, we established a pixel-by-pixel correlation between ADC and BBB permeability values for the ipsilateral side of both cerebral cortex and subcortex at the two different time points under study. Scatterplots shown in **Fig. 4** indicate that there are more pixels with increased permeability (K_i_>0.001 ml/g-min) at 48 h than at 3 h, supporting data shown in **Fig. 2**. We observed a marked heterogeneity in the population of pixels with high K_i_ values and either reduced or normal ADC values. At 3 h, we found a large number of pixels with very low K_i_ (<0.001 ml/g-min) and a wide range of ADC values, from low to normal (higher than 80% of the contralateral ADC average). Between 3 and 48 h, we observed a shift in the population of pixels with low K_i_ and either low or normal ADC toward a higher K_i_ value (**Fig. 4**). We identified three main different populations of pixels within each brain area in the ipsilateral side: high K_i_ (>0.001 ml/g-min) and low ADC (lower than 80% of the contralateral ADC average), high K_i_ and normal ADC, and normal K_i_ and low ADC. We calculated the area for each population of pixels (**Fig. 5**). We built a color-coded K_i_-ADC map where the three main populations of pixels with different K_i_ and ADC values are spatially represented for both time points (**Fig. 5A and 5B**). For the cerebral cortex and subcortex, we found a dramatic significant increase between 3 and 48 h in the areas with high K_i_ and either low or normal ADC (**Fig. 5C and 5D**). In cerebral cortex, we did not find a statistically significant reduction in the areas with normal K_i_ and low ADC over time (**Fig. 5C**). However, in the subcortical regions there was a significant decrease in the areas with reduced ADC and normal K_i_ values (**Fig. 5D**).

## Discussion

We report a MRI-based pixelwise analysis of BBB permeability and ADC values in a clinically relevant model of ischemic stroke. These data indicate that BBB breakdown is not only restricted to areas with reduced ADC. Furthermore, areas with low ADC values do not always have increased BBB opening. Having both spatial and intensity data provides a more accurate picture of the BBB disruption. Adding ADC information more accurately characterizes the state of the tissue pixel by pixel. Such unique information can be used to assess the role of ischemia (ADC) on vascular integrity (BBB permeability). With this information, the benefits of a treatment can be more clearly assessed.

The increase in tissue area in regions with high K_i_ and low ADC suggest that these are the core areas that are most severely damaged and are unlikely to be salvageable. A region with high K_i_ and normal ADC has growth but to a lesser degree, and some return of function may be possible. This might suggest secondary permeability alterations in areas surrounding the ischemic core, while cellular viability is not compromised. However, part of the region with preservation of the BBB integrity, in spite of an ischemic insult that causes low ADC, had some chance of recovery. We found that sections of this region were less affected at 48 h; this reduction may also be interpreted as showing removal of edema fluid, which may be possible when the capillary is intact. In such a case, agents that act to preserve capillary function may provide the basis for cellular recovery even in the event of injury to other cells.

Our data are in line with previous reports indicating that over time, the amount of tissue lost increases and the cerebral cortex accounts for most of the brain tissue that eventually becomes infarcted [14], [28], [29]. It is now well documented that neuroinflammatory mechanisms are responsible for the neuronal death in the ischemic penumbra, a potentially recoverable tissue surrounding the infarct core [30]. Since the pixels with high K_i_ and normal ADC were surrounding the core, this region most likely represent penumbra, which is consistent with inflammatory BBB opening.

Our present data indicate that in the cerebral cortex the extravascular Gd-DTPA enhancement, indicative of BBB breakdown, is observed in large areas and the BBB permeability coefficients at 48 h are not significantly different from 3 h. Unlike the cortex, in the subcortical areas we found significant differences in both the degree and area of leakage between 3 and 48 h. The reason(s) for these regional differences is not presently known, but could be related to the degree of tissue infarction. By 48 h of recirculation, much of the ischemic core (mainly subcortex) had undergone cell death, thus the BBB is disrupted, and the contrast agent could accumulate resulting in high K_i_ values. A recent study utilizing a rat model of 90 min of transient focal cerebral ischemia found that there is an inverse correlation between K_i_ and ADC values [31], which suggest that the degree of ischemic brain damage may influence the extent of BBB permeability.

Detection of BBB opening using D-CEMRI largely depends on the delivery of the paramagnetic contrast agent to the affected areas of the brain. Thus, it may be possible that we missed the BBB damage in areas with marked ADC hypointensities due to inefficient delivery of Gd-DTPA. This could have been the result of disturbances in cerebral blood flow (CBF) in the affected region. Whether or not regional reductions in CBF determining the delivery of Gd-DTPA may be a confounder in our results is difficult to recognize based on our data.

An ADC threshold set at approximately 80% of the normal ADC has been found to correlate well with the loss of ATP and breakdown of energy metabolism in an animal model of temporary focal cerebral ischemia [32]. However, using proton MR spectroscopic imaging along with several MRI techniques including ADC mapping, a previous human study showed that there is a large metabolic heterogeneity in tissue damage inside ischemic areas showing similar decreased mean ADC values [19]. This suggests that areas with similar reduction in ADC values have different stages of ischemic injury, and possibly degree of BBB permeability changes.

We used data from two groups: one at 3 h and the other at 48 h. This limited this study to two time points after recirculation, representing the early (3 h) and delayed BBB disruption (48 h) in this rat stroke model [11]–[13]. As confirmed statistically, we observed low variability in the studied parameters at these time points between the two groups subjected to MCAO. Thus, changes seen between 3 and 48 h reflect progression of ischemic injury. The homogeneity of the groups allowed for the analysis of the correlations between ADC and BBB alterations at 3 and 48 h in these two groups even though they are formed by different animals. It would have been ideal to perform a longitudinal study using the same animals. We believe that future studies have to be conducted to include other time points to better characterize the temporal changes in water homeostasis and its relation to cell death after ischemia.

Based on data from **Figs. 4** and **5**, we hypothesized that there might be a model representing the relationships between K_i_ and ADC with the temporal evolution of the infarct in this rat stroke model. A previous study has utilized the generalized linear modeling (GLM) technique for finding structural correlations between parameters obtained by multimodal MRI in human cerebral ischemia [33]. To obtain a simple predictive model of tissue fate in cerebral cortex, we correlated ADC and BBB permeability for the two time points assuming that data are independently and identically distributed. Using GLM over all pixel values, we obtained the following linear structural equation for predicting K_i_ values at 48 h in the cerebral cortex:
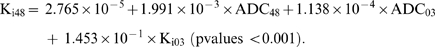



This model indicates that it is possible to predict the BBB permeability in the cerebral cortex at the pixel level at 48 h of reperfusion, and thus to build the permeability map at this time point from the ADC and K_i_ values at 3 h and the ADC at 48 h in the cerebral cortex.

We then tested this model for the subcortex area and found that the coefficient for ADC at 3 h was not statistically significant indicating that ADC at 3 h is not a component of the corresponding structural model. Thus, the equation for predicting K_i_ values at 48 h for the subcortical regions reduces to the following equation:
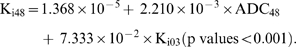



The differences in these two structural equations for cortical and subcortical areas clearly indicate that the nature of correlations between BBB permeability and edema in these two regions are different.

**Figure 6:**
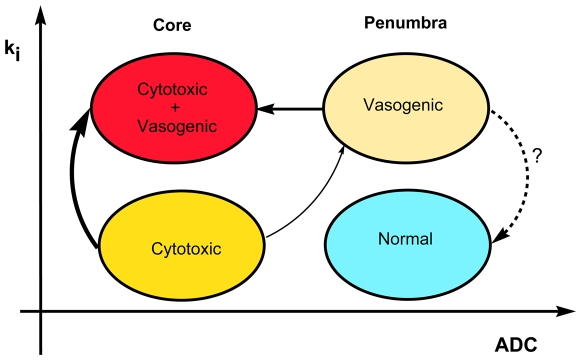
Proposed model showing possible transitions between different states of tissue injury during the progression of ischemic stroke. Cytotoxic edema is characterized by low ADC values with preserved BBB function (normal K_i_), while vasogenic edema is identified by high K_i_ values. The core of the infarct display high K_i_ and low ADC values (a mixture of cytotoxic and vasogenic edema). Based on data from the merged ADC+K_i_ maps (Fig. 5), the main transition occurs from areas with cytotoxic edema to areas with both cytotoxic and vasogenic edema (core of the infarct), which is depicted with a thicker arrow in the schematic.

Over time, we observed reduction in the area with low ADC and normal K_i_ (cytotoxic edema) and increase in regions with low ADC and high K_i_ (cytotoxic+vasogenic edema), suggesting that early hypoxia with preserved BBB function grows into core tissue unlikely to recover. On the other hand, pixels with normal ADC and high K_i_ (vasogenic edema) showed only moderate growth. When the BBB is disrupted and the cell has not swollen yet, the vasogenic edema occurs. However, when the cell begins to swell with or without BBB injury, cytotoxic edema occurs. Generally, there is some combination of both with one predominating. A proposed model representing transition between different states of tissue is shown in **Fig. 6**.

While there is consensus that a drop in ADC is a harbinger of cell death, the implications of changes in BBB permeability are less clear. Combining ADC with K_i_ provides insights into the role of permeability. Assuming that high K_i_ with normal ADC equates with inflammatory BBB opening, pixels with these characteristics have the potential to recover. However, when ADC is low, suggesting more severe hypoxia, the potential for evolution into core-like pixels appears to be greater. Although the variability in the individual groups was small, further studies will be needed in the same animal over multiple times to validate a predictive model. Finally, the ability to separate vasogenic and cytotoxic components, even if not totally precise, will be very informative in testing drugs and will have us closer to the ability to use multimodal MRI to test drugs in clinical trials.

## Materials and Methods

### Rat Model of Focal Cerebral Ischemia

All animal studies were approved by the University of New Mexico Animal Care and Use Committee and conformed to NIH guidelines for use of animals in research. Sixteen Wistar rats weighing 280–300 g were used in this study. The rat focal cerebral ischemia model has been described in detail previously [4], [12]. In brief, the right hemisphere focal cerebral ischemia was induced in animals by inserting a nylon filament through the external carotid artery (ECA) in order to occlude the middle cerebral artery. After 2 h of ischemia, the filament was removed from the ECA to allow reperfusion.

### Magnetic Resonance Imaging (MRI)

For the MRI study, two groups of rats were used: the first group consisting of 8 rats was studied 3 h after the reperfusion while the second group of rats was studied at 48 h (n = 8). Prior to MRI, rats were anesthetized with 2.5% isoflurane at 1 L/min N_2_O/O_2_ (70∶30) flow under spontaneous respiration. Heart rate and respiration rate were continuously monitored during scanning and maintained within normal values. MR imaging was performed on a 4.7T horizontal bore Biospec^®^ dedicated research MR scanner (Bruker Biospin, Billerica, MA), equipped with 500 mT/m (rise time 80–120 µs) gradient set (for performing small animal imaging) and a small bore linear RF coil (ID 72 mm).

### ADC Measurements

First, T2 weighted and diffusion weighted imaging (DWI) were performed with the following parameters; T2 weighted-2D RARE (Rapid Acquisition with Relaxation Enhancement), TR/TE 4000/65 ms, FOV 3.2 cm×3.2 cm, slice thickness 2 mm, slice gap 1 mm, number of slices 2, matrix 256×128, number of averages 5, receiver bandwidth 100 kHz; DWI - 2D Diffusion weighted RARE, TR/TE 2000/31.2 ms, FOV 3.2 cm×3.2 cm, slice thickness 2 mm, slice gap 1 mm, number of slices 2, matrix 64×64, number of averages 22, receiver bandwidth 100 kHz, d = 5 ms, D = 20 ms, b value of 0 and 900 s mm^−2^, diffusion gradient along the slice select direction. From DWI images, pixel-by-pixel maps of ADC of water were estimated [4]. We utilized an ADC threshold defined as pixels with ADC values lower than 80% of the mean contralateral hemisphere values on the ADC map [34]. The slices containing the lesion were identified from the T2 weighted structural images and same slice location was prescribed for all the subsequent MR protocols. We carefully separated the cerebral cortex and subcortex by registering the anatomical images (T2-w) into ADC and BBB maps and demarcating the brain areas in each slice. At 48 h of reperfusion, tissue damage seen in ADC maps was confirmed using the TTC staining. Two-mm thick brain slices were incubated at 37°C for 30 min in a 2% solution of TTC as described in detail in our previous study [11].

### BBB Permeability Measurements

BBB permeability was studied with a modification of D-CEMRI [21], [25] as described in our recent studies [4], [35]. Rats were injected 0.1 mmol/kg of gadolinium-diethylene-triamine-pentaacetic acid (Gd-DTPA, MW = 938 Da; Bayer Healthcare) as a bolus into the femoral vein via an indwelling catheter, followed by imaging with rapid T1 mapping protocol, collecting 14 images over 45 minutes. We visually checked the linearity of the plots [21] for the whole duration of the T1 imaging. The following optimized MR imaging parameters were used for this protocol: axial plane, two-dimension, inversion recovery-spin echo-echo planar imaging (IR-SE-EPI), TR/TE 8 s/19.4 ms, FOV 4.0 cm×4.0 cm, slice thickness 2 mm, slice gap 1 mm, number of slices 2, matrix 64×64, number of averages 2, receiver bandwidth 250 kHz. Non slice selective magnetization inversion was performed using a hyperbolic secant (sech) RF pulse with pulse width 4 ms. The T1 mapping specific parameters for this protocol were: time for inversion (TI) = (100+600×n) ms where n = 0, 1, 2, …12, number of TI points (n) is 13. The total scan time was 3 minutes and 12 seconds for each time point. The MRI protocol parameters for rapid T1 mapping were optimized for accuracy of T1 relaxation time estimate in a single, normal rat brain (control) prior to including that animal in the study.

### MRI Data Analysis

Data processing was performed with custom software written in MATLAB (Mathworks, Natick, MA). Image analysis was performed using ImageJ (NIH, Bethesda, MD) and MRVision (Winchester, MA) software packages.

Permeability maps were constructed using time series data obtained by D-CEMRI, and a pixel by pixel method based on a compartmental modeling technique first proposed by Patlak [36] and used by others [4], [17], [21]. In brief, the method is based on the leakage of the contrast agent from plasma compartment into brain compartment through BBB resulting in the change of the MR signal intensity. The rate of changes in MRI signal intensity relates to BBB permeability (K_i_). Optimal value for K_i_ for each pixel was calculated by fitting the Patlak model equations into the time series of the pixel intensity employing nonlinear regression analysis. Then the K_i_ value of each pixel is color-coded to construct a BBB permeability map, where pixels with high intensity color represent high BBB permeability locations in the brain. Based on a previous report [27], we considered K_i_ values higher than 0.001 ml/g-min as increased permeability. For correlation analysis between ADC and K_i_ maps, we performed image registration using image-processing toolbox of Matlab.

### Statistical Analysis

The data were subjected to statistical analysis for two related purposes: description and inference. For descriptive statistics, data were separated into four different sets; ADC for cerebral cortex and subcortical areas, permeability for cerebral cortex and subcortical areas by accumulating pixel-based data of each region into a different set. Then **R** statistical computing environment (**R** Development Core Team, 2007) was used to represent the histogram of data, and analyze the corresponding distributions [37]. This was followed by inferential statistics using a generalized linear model [38] in **R** environment to derive structural equations defining correlations between data sets. Other statistical comparisons between data sets were made based on the representation of mean±SEM. Student's t-test (parametric data) and Mann-Whitney test (non-parametric data) were used for the statistical analyses. The increase in the percentage of pixels with high K_i_ (>0.001 ml/g-min) between 3 and 48 h was analyzed running a 2×2 contingency table with χ^2^ statistics. A p value less than 0.05 was considered statistically significant.
